# Non-traumatic perforation of the jejunum in a human immunodeficiency virus-infected patient receiving combination antiretroviral therapy: A case report

**DOI:** 10.1097/MD.0000000000018163

**Published:** 2019-12-10

**Authors:** Yi-Chien Lee, Chien-Chun Chiou, Jann-Tay Wang, Yi-Chun Yang, Shao-Hsien Tung, Szu-Min Hsieh

**Affiliations:** a Department of Internal Medicine, Fu Jen Catholic University Hospital; b School of Medicine, College of Medicine, Fu Jen Catholic University, New Taipei City; c Department of Dermatology, Ditmanson Medical Foundation Chia-Yi Christian Hospital, Chia-Yi; d Department of Internal Medicine, National Taiwan University Hospital and National Taiwan University College of Medicine, Taipei; e Institute for Infectious Diseases and Vaccinology, National Health Research Institutes; f Department of Nursing, Ditmanson Medical Foundation Chia-Yi Christian Hospital, Chia-Yi, Taiwan.

**Keywords:** bowel perforation, cytomegalovirus, immune reconstitution inflammatory syndrome, *Mycobacterium avium* complex

## Abstract

**Rationale:**

Non-traumatic bowel perforation caused by cytomegalovirus (CMV) and *Mycobacterium avium* complex (MAC) infections has become rare among patients with acquired immunodeficiency syndrome (AIDS) in the era of combination antiretroviral therapy (cART); however, CMV-associated and MAC-related immune reconstitution inflammatory syndrome (IRIS) has subsequently emerged owing to the wide use of integrase inhibitor-based regimens. Here we report a case of spontaneous perforation of the jejunum in a patient with human immunodeficiency virus (HIV) infection with good compliance to cART.

**Patient concerns:**

A 32-year-old HIV-infected man developed CMV disease and DMAC infection, as unmasking IRIS, 3 days after the initiation of cART. After appropriate treatment for opportunistic infections, intermittent fever with enlarged lymph nodes in the abdomen occurred as paradoxical IRIS. The patient was administered prednisolone with subsequent tapering according to his clinical condition.

**Diagnoses:**

Unexpected perforation of hollow organ during the titration of steroid dose with clinical presentations of severe abdominal pain was diagnosed by chest radiography.

**Interventions:**

He underwent surgical repair with peritoneal toileting smoothly.

**Outcomes:**

He was discharged well with a clean surgical wound on post-operative day 10.

**Lessons:**

Bowel perforation may be a life-threatening manifestation of IRIS in the era of cART. Steroids should be avoided, if possible, to decrease the risk of bowel perforation, especially in IRIS occurred after opportunistic diseases involving the gastrointestinal tract.

## 1 Introduction

Non-traumatic perforation of the gastrointestinal tract is scarcely seen both in the general population and in patients with HIV infection.^[1]^ The etiologies include immune-mediated diseases (e.g., Crohn disease), infections (e.g., CMV, Mycobacterium spp.), drugs (e.g., indomethacin, steroids), metabolic disorders, vascular insufficiencies and neoplasms.^[2]^ CMV, a DNA virus belonging to the group of herpes viruses,^[3]^ may lead to injuries in specific organs, including the retina, respiratory system, central nervous system, and gastrointestinal tract,^[4]^ in patients with AIDS. The most frequently affected region of the gastrointestinal tract is the colon (47%), followed by the duodenum (21.7%), stomach (17.4%), esophagus (8.7%), and small intestine (4.3%).^[5]^ Furthermore, the main location of bowel perforation in patients with AIDS is the colon (53%), followed by the distal ileum (40%) and the appendix (7%).^[6]^

Nontuberculous mycobacteria varying in pathogenicity are rather ubiquitous in the natural environment,^[7]^ and MAC from water, soil, and food can cause infections in immunocompromised hosts through inhalation and ingestion.^[8]^ This bacterium commonly causes disseminated MAC (DMAC) infection in HIV-positive patients with CD4 lymphocyte counts <50 cells/μL. Moreover, MAC infection can also involve the whole gastrointestinal tract with various appearances, including multiple raised nodules or normal-appearing mucosa in the stomach on endoscopy, thickened or edematous mucosal folds in the small bowel, and flattened mucosa in the colon on colonoscopic examination.^[9]^

IRIS, also known as immune reconstitution disease, has been much more frequently encountered in clinical settings after the initiation of cART worldwide, and a wide range of pathologies have been disclosed.^[10]^ Notwithstanding, all of the aforementioned HIV-associated gastrointestinal diseases rarely exhibit perforation of the involved hollow organs, implying the emergence of IRIS. Herein, we describe a HIV-positive patient with good adherence to cART who developed spontaneous perforation of the jejunum.

## 2 Case report

A 32-year-old man with HIV infection initially presented with oral candidiasis and latent syphilis. His baseline plasma HIV RNA load (PVL) was 1,110,000 copies/mL with a CD4 count of 25 cells/μL. Half a month after the HIV diagnosis, he started cART with dolutegravir/abacavir/lamivudine, and was admitted because of intermittent fever and diarrhea 3 days after the initiation of cART. Two-week intravenous ganciclovir was prescribed on the 4th hospital day with subsequent 10-day oral valganciclovir because CMV gastritis/duodenitis and colitis were diagnosed by biopsy via panendoscopy and colonoscopy after admission, respectively. Additionally, anti-MAC therapy with imipenem, amikacin, clarithromycin, and ethambutol was started on the 12th hospital day when MAC was isolated from cultures of the sputum, blood, and colon specimens. After 1 month of cART, his PVL decreased to 315 copies/mL and CD4 count increased to 33 cells/μL. However, intermittent fever persisted despite continuation of appropriate anti-MAC therapy for more than 2 weeks. Computed tomography of the abdomen on the 28th hospital day demonstrated splenomegaly with multiple enlarged mesenteric and para-aortic lymph nodes; however, no other significant pathogens were identified. Thus, the patient was started on twice-daily 15-mg prednisolone on the 31st hospital day under the tentative diagnosis of IRIS, and steroids were gradually tapered down. In the following days, his clinical condition became more stable and his fever subsided. He was discharged home on the 47th hospital day.

**Figure 1:**
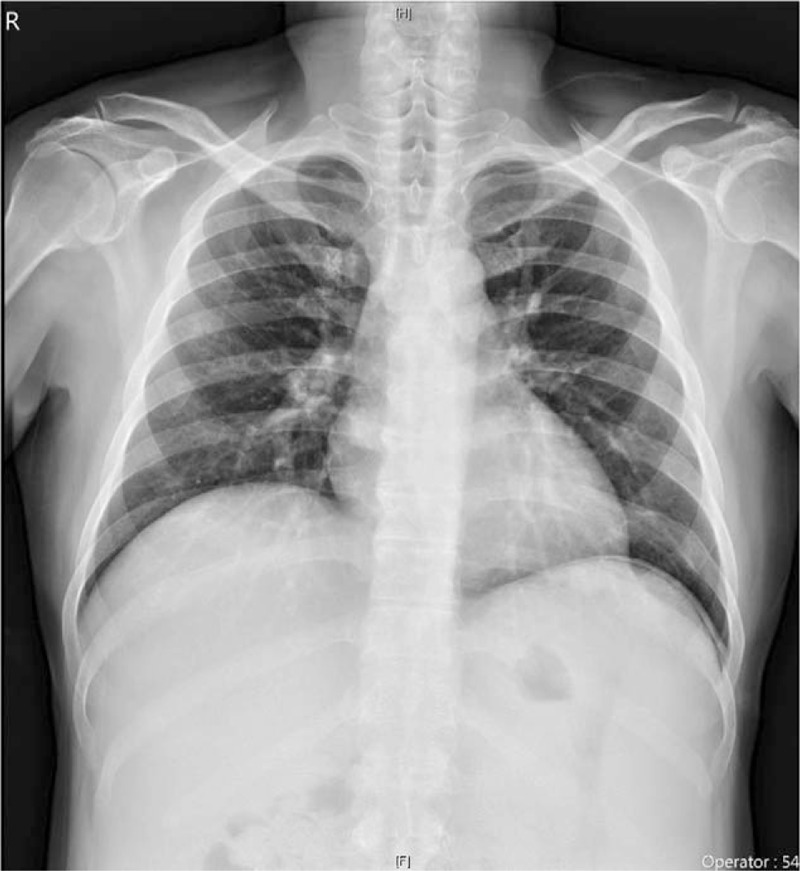
Chest radiography. Chest X-ray demonstrating the presence of free air below left side hemidiaphragm.

Nevertheless, 3 days after discharge, the patient was readmitted to the hospital for severe abdominal pain with nausea, poor appetite, and a spiking fever of 38.7°C. At presentation, his blood pressure was 121/78 mmHg and pulse rate was 138 beats/min. On physical examination, he showed an ill appearance with hypoactive bowel sound, rebound tenderness on the abdomen, and abdominal muscle guarding. Laboratory investigations showed a white blood cell count of 3000 cells/μL, hemoglobin level of 8.1 g/dL, platelet count of 194,000/μL, and elevated alanine amino transferase level of 102 U/L; other laboratory parameters were otherwise normal. Chest radiography revealed the presence of free air below the left-sided hemidiaphragm (Fig. 1), and perforation of the hollow organ was highly suspected. A surgeon was consulted immediately, and the patient was subsequently taken to the operating room. During the exploratory laparotomy, 1 small perforation was found 40 cm away from the ligament of Treitz in the jejunum, and surgical repair with peritoneal toileting was performed without problems. Enteral feeding was established on post-operative day 5, and cART with anti-MAC regimen was resumed later. He was discharged well with a clean surgical wound on post-operative day 10. After discharge, he received regular outpatient management for up to 30 months with a good adherence to cART, and undetected PVL with gradual recovery of CD4 count was observed.

## 3 Discussion

We presented here a complicated case. Our patient with AIDS initially presented with oral candidiasis. Intermittent fever developed 3 days after the initiation of cART. CMV disease and DMAC infection were diagnosed subsequently, which are compatible with unmasking IRIS.^[10,11]^ However, after completion of at least 3-week of anti-CMV treatment with anti-MAC regimen for more than 5 weeks, intermittent fever with mesenteric and para-aortic lymphadenopathy supervened, suggesting the occurrence of paradoxical IRIS.^[10,11]^ Furthermore, perforation of hollow organ unexpected occurred during steroid tapering. This is a rare case of spontaneous perforation of the jejunum in a patient with AIDS with CMV disease and DMAC infection who was compliant with cART. Unfortunately, no specimen was taken for pathological examination during the operation for bowel perforation; however, CMV- and MAC-associated IRIS and concurrent use of steroids might have contributed to the event.

In HIV-positive patients, most cases of intestinal perforation due to CMV infection occur in the advanced stage without a well-suppressed viral load;^[4,6]^ however, a few case reports demonstrated presentations of bowel perforation secondary to CMV-associated IRIS with prescription of early cART.^[12]^ Additionally, the involved organ in MAC-associated IRIS can be variable, but is most often the lymph nodes, liver, gastrointestinal tract, and even the lungs.^[13]^ Different clinical phenomena of IRIS associated with MAC implicating intra-abdominal regions, including retroperitoneal abscess, colitis, mesenteric lymph node involvement, chylous ascites, and intestinal obstruction, have been previously reported.^[14]^ There have been cases of intestinal perforation caused by *Mycobacterium tuberculosis*-related IRIS;^[15]^ however, there is only 1 similar case report published in 2008.^[16]^

With the introduction of cART in 1996, the incidence rate of opportunistic infections, including CMV disease, DMAC infection, and AIDS-related death, dramatically declined in HIV-positive patients.^[17]^ However, unexpected clinical events, known as IRIS, including paradoxical deterioration of treated opportunistic infections or unmasking of antecedently undetected untreated infections, might occur during the initial months, or even years, of cART.^[18]^ Generally, making a diagnosis of CMV-related or MAC-associated IRIS is difficult because IRIS has remained a clinical syndrome caused by widely diverse pathogens and variable disease presentations. Our patient fulfilled those presumed criteria,^[19]^ as mentioned above. Furthermore, several risk factors predisposed our patient to the development of IRIS, including rapid decrease in PVL due to the use of an integrase inhibitor-based regimen, lower baseline CD4 count before cART initiation, and an anti-retroviral naïve status. In contrast, cART, including integrase inhibitors, is currently recommended as a first-line therapy for HIV-infected patients, even in the advanced stage of AIDS,^[20]^ because of its potent effect and good tolerability.^[21]^ Yet, potential harmful results of IRIS may develop owing to fast decreasing in plasma HIV viral load^[22]^; thus, physicians should be aware of the emergence of IRIS during the treatment of HIV infection with integrase inhibitor-based regimens.

Most cases of IRIS are benign and self-limited, although severe cases with eventual mortality have also been described.^[14]^ As cART is crucial for improving immune function, the application of this treatment without interruption is recommended. Whether the adjunctive use of corticosteroid offers favorable prognosis among HIV-infected patients with IRIS remains controversial; however, its use for patients with life-threatening IRIS, for example, massive inflammation in the central nervous system, is indicated.^[23]^ Notwithstanding, administration of systemic corticosteroid will lead to a variety of possible harmful outcomes, including infective complications,^[24]^ glucose intolerance, increasing in blood pressure level, reduction in bone mineral density, and gastrointestinal ulceration.^[11]^ Overall, the use of corticosteroids may increase the risk of gastrointestinal perforation by 40%.^[25]^ In our patient, acute onset of abdominal pain with intestinal perforation occurred during the process of steroid tapering. Therefore, alertness to symptoms and signs of bowel perforation with judicious use of steroids will be helpful among HIV-infected patients with presumed IRIS following opportunistic diseases of the gastrointestinal tract.

## 4 Conclusion

The incidence of CMV disease and DMAC infection will continuously decline with increased accessibility of cART; however, CMV- or MAC-associated IRIS will possibly be encountered much more commonly as integrase inhibitor-containing therapy becomes widely administered worldwide. Although no pathologic evidence could verify the etiology of bowel perforation in the presented case, CMV-related, MAC-associated IRIS and concurrent use of steroids might have contributed to the event. Hence, steroids should not be prescribed, if possible, to lower the risk of gut perforation, particularly in patients acquiring opportunistic infections involving the gastrointestinal tract with subsequent development of IRIS.

## Author contributions

**Conceptualization:** Yi-Chien Lee, Chien-Chun Chiou, Jann-Tay Wang.

**Data curation:** Yi-Chun Yang, Shao-Hsien Tung.

**Investigation:** Yi-Chun Yang, Shao-Hsien Tung.

**Methodology:** Jann-Tay Wang, Szu-Min Hsieh.

**Validation:** Jann-Tay Wang.

**Writing – original draft:** Yi-Chien Lee, Chien-Chun Chiou.

**Writing – review & editing:** Yi-Chien Lee, Chien-Chun Chiou, Szu-Min Hsieh.
